# Are Emergency Medicine Residents Provided Adequate Training in Patient-Centered Communication?: A Resident Survey

**DOI:** 10.51894/001c.6782

**Published:** 2018-04-27

**Authors:** Kimberly Stillman, Jesse Kellar

**Affiliations:** 1 Emergency Medicine Resident PGY-4 Lakeland Health Emergency Medicine Residency; 2 Assistant Program Director Lakeland Health Emergency Medicine Residency

**Keywords:** resident training, patient-centered communication

## Abstract

**CONTEXT:**

Emergency Medicine residents frequently face challenging communication situations. Little is known regarding resident comfort level and amount of training received in managing these types of patient care scenarios. The purpose of this study was to measure the relationships between Emergency Medicine resident comfort levels, reported amount of patient-centered communication training received and correlation between amount of GME training and comfort levels when handling difficult situations in emergency departments.

**METHODS:**

In 2016, the authors used the Council of Emergency Medicine Residency Directors (CORD) listserv to disseminate an online survey to Emergency Medicine residents. Survey content came from the Patient Centered Communication subsection of The American Board of Emergency Medicine Milestones. This survey included five different patient scenarios.

**RESULTS:**

There were a total of 306 completed surveys. Residents rated their comfort level as most comfortable in scenarios regarding exhibiting empathy and least comfortable when providing bad news to patients or dealing with drug-seeking patients and difficult family members. Training was most prevalent in the areas of exhibiting empathy and giving bad news and lowest in managing drug-seeking patients and difficult patients.

**CONCLUSIONS:**

This survey revealed that Emergency Medicine residents do not consider themselves generally comfortable in multiple communication scenarios and that the amount of training received in these areas is often lacking during residency. A statistically significant positive correlation existed between comfort level and amount of graduate medical training in most areas. Results suggest that increasing the amount of communication training during residency may be of benefit in influencing how comfortably residents handle difficult patient scenarios.

## INTRODUCTION

Current literature regarding patient-centered communication (PCC) skills training during residency is lacking any clear consensus on the best way to educate residents. Additionally, the literature available relies on a limited body of research investigating the difficult communication situations in which residents often find themselves. It has, however, been demonstrated, that residents benefit when they receive specific PCC training in addition to technical training.^1–4^ Different PCC training modalities have been examined, including simulation labs, week-long courses, and standardized patient encounters.^3,5^ There is evidence that Emergency Medicine (EM) residents who have undergone communication skills training programs are more confident and consistently receive greater patient satisfaction scores and fewer patient complaints.^3^

Emergency Medicine residents are required to reach milestones that show competence in a variety of areas, one of which is “Patient-Centered Communication.”^6^ (Appendix 1) In response, the authors created a survey to identify specific areas of PCC skills in which residents might feel stronger and weaker. The authors designed the survey to determine how much PCC training residents had previously received at time of survey. Before the study, the authors had hypothesized that most residents would respond that they had not received adequate PCC training in these areas and would not be entirely comfortable in these types of scenarios.

**Appendix 1:**
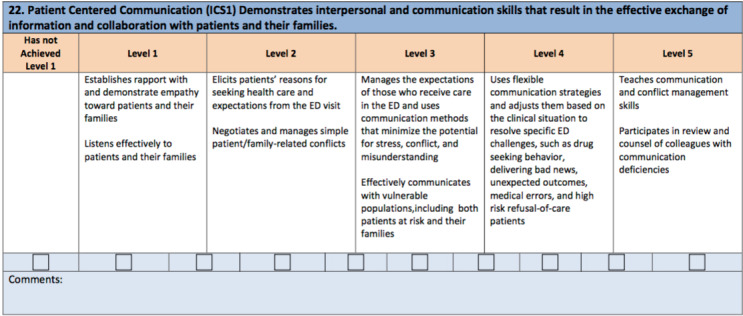
The American Board of Emergency Medicine Milestones on Patient Centered Communication

## METHODS

An online survey of EM residents was disseminated using the Council of Emergency Medicine Residency Directors (CORD) listserv that includes 194 EM program directors.^7^ The program directors were asked to forward the survey to their respective residents to investigate the comfort level and amount of PCC training received in handling common challenging communication situations in their emergency department. All survey responses were anonymous. Survey links were sent on May 15^th^, 2015 and the survey closed on June 15^th^, 2015. Prior to data collection, the project received an exempt approval by the Lakeland Health Institutional Review Board.

Survey scenarios came directly from The American Board of Emergency Medicine’s (ABEM) established EM Resident Milestones under the subsection entitled “Patient Centered Communication.”^6^ (Appendix 1) The specific scenarios that were chosen included: *a) Giving patients bad news, b) Dealing with drug-seeking patients, c) Handling difficult family members/friends of a patient, d) Managing high-risk patients who refuse medical care, and e) Exhibiting empathy towards patients.*

The first five questions of the survey described some of these PCC scenarios and asked respondents first how comfortable there were with their communication skills in a given scenario, and second, how much prior PCC training they had received to manage the scenario. A Likert-type scale of 1-5 was used to rate comfort level with “1” correlated with “Very Uncomfortable” and a “5” response being “Very Comfortable.” Similarly, a 1-to-5 numerical Likert scale was used to rate the amount of PCC training received with qualifiers next to each number. For this item, a “1” correlated with “No Training” and “5” being “An Extensive Amount of Training.”

### Analyses

The survey results were analyzed using standard descriptive statistics, such as Mean with Standard Deviation and Frequency (Percentage) for each question. To examine for any significant correlations between respondents’ comfort level ratings and their amount of PCC training for each scenario, a series of Pearson’s Chi-Square tests procedures were run. A Coefficient Alpha P value of <.05 was considered statistically significant. All analyses were completed using SPSS version 22 software.^8^

## RESULTS

The authors received a total of 306 surveys. The respondent characteristics are shown in Table 1. It is important to note that less than 2% of data concerning these survey items were missing from sample respondents.

**Table 1:** Respondent Characteristics (306 Emergency Medicine Residents)

**Characteristic**	**n**	**Percent**
**Gender**		
Male	177	57.8
Female	127	41.5
Missing	2	0.6
**Community-based vs. University-based training**		
Community	125	40.8
University	181	59.2
**Size of Residency**		
Small (< 24 residents)	55	17.9
Medium (25-36 residents)	135	44.1
Large (>37 residents)	110	35.9
Missing	6	1.9
**Year of Training**		
Junior Resident (1^st^ or 2^nd^ year)	191	62.4
Senior Resident (3^rd^ or 4^th^ year)	113	36.9
Missing	2	0.6

- 127/304 female (41.8%), 177/304 male (58.2%)
- 125/306 community-based (40.8%), 181/306 (59.2%) university-based
- small (<24 residents) 55/300 (18.4%), medium (25-36 residents) 135/300 (45.2%), large >37 residents 110/300 (36.4%)
- 191/304 (62.8%) junior residents, 113/304 (37.2%) senior residents

### I. Comfort and Training Level Data in Communication Scenarios

When comparing comfort levels among residents during the five PCC scenarios, results showed that the mean comfort level in all areas were between 3.6 and 4.4 (Table 2). Respondents felt most comfortable showing empathy towards patients (Mean 4.40; SD 0.918), followed by giving bad news (Mean 4.04; SD 0.889), refusal of care (Mean 3.75; SD 0.961), dealing with drug-seeking patients (Mean 3.669; SD 0.997), and dealing with difficult patients (Mean 3.667; SD 0.958). There was a relative trend towards senior residents (i.e., PGY 3 and PGY4) feeling more comfortable in all PCC scenarios except in the category of exhibiting empathy (Table 3).

**Table 2:** Resident Comfort Levels

**Resident Comfort Level**			
**Scenario**	**Mean**	**SD**	
Exhibiting Empathy (N=303)	4.40	0.92	
Giving Bad News (N=305)	4.05	0.89	
Refusal of Care (N=305)	3.75	0.96	
Drug-Seeking (N=306)	3.67	1.00	
Managing Difficult People (N=306)	3.67	0.96	
**Scale:** 1-very uncomfortable, 2-somewhat uncomforable, 3-neither comfortable, 4-somewhat comfortable, 5-very comfortable			
			
**Amount of Resident Training**			
**Scenario**	**Mean**	**SD**	
Giving bad news (N=285)	2.95	0.90	
Exhibiting Empathy (N=285)	2.76	0.84	
Refusal of Care(N=289)	2.62	0.91	
Drug Seeking (N=296)	2.53	0.84	
Managing Difficult People (N=301)	2.50	0.83	
**Scale:** 1-No training, 2-minimal training, 3-some training, 4-significant training, 5-extensive training			

**Table 3:** Junior vs. Senior Resident Comfort Levels

**Comfort level by PGY year**	** **	**Mean**	**SD**
			
**Exhibiting Empathy (N=304)**	** **	**4.4086**	**0.9179**
PGY 1-2 (N=189)		4.4286	0.9348
PGY 3-4 (N=112)		4.3750	0.8917
			
**Giving Bad News (N=303)**	** **	**4.0462**	**0.8903**
PGY 1-2 (N=190)		3.9368	0.9350
PGY 3-4 (N=113)		4.2301	0.7793
			
**Refusal of Care (N=303)**	** **	**3.7525**	**0.9567**
PGY 1-2 (N=190)		3.6789	1.0116
PGY 3-4 (N=113)		3.8761	0.8466
			
**Drug Seeking (N=304)**	** **	**3.6678**	**1.0007**
PGY 1-2 (N=191)		3.5288	0.9883
PGY 3-4 (N=113)		3.9027	0.9817
			
**Managing Difficult People (N=304)**	** **	**3.6645**	**0.9609**
PGY 1-2 (N=191)		3.5707	0.9646
PGY 3-4 (N=113)		3.8230	0.9376

Comparing the amount of prior PCC training among residents in all communication scenarios, the mean amount of training in all areas fell between 2.50 and 2.95 (Table 2). Residents received the most training in giving bad news to patients and families (Mean 2.95; SD 0.835), followed by empathy (Mean 2.76; SD 0.899), refusal of care (Mean 2.61; SD 0.905), dealing with drug seeking patients (Mean 2.52; SD 0.843), and dealing with difficult family/friends of patients (Mean 2.50; SD 0.831).

### II. Does comfort level correlate with amount of PCC training?

A series of Pearson product-moment correlation coefficient were generated to examine the relationship between residents’ comfort levels and their amount of PCC training for each scenario. Overall, there was positive correlation between comfort level and amount of PCC training for all situations except for empathy (Table 4). The four of five areas in which there was a positive correlation were each statistically significant with a P value of < 0.001. The strongest correlation was in the area involving refusal of care (r = +.378, n = 305, p < 0.001), followed by dealing with drug-seeking behavior (r = +.287, n = 306, p < 0.001), dealing with difficult family/friends (r = +.215, n = 306, p < 0.001), and giving bad news (r = +.193, n = 305, p < 0.001). The product coefficient when examining the relationship between comfort level of demonstrating empathy and the amount of training was not significant at r of +.018, n = X303, and P value of 0.767.

**Table 4:** Correlations between comfort level and amount of PCC training received

**Comfort level with amount of training**	**P value**
Exhibiting Empathy (N=303)	0.767
Giving Bad News (N=305)	0.001
Refusal of Care (N=305)	0.001
Drug Seeking (N=306)	0.001
Managing Difficult People (N=306)	0.001

### III. Learning Method Preference Data

Residents were asked what their preferred method of learning in residency was outside of physically working in their emergency department. (Table 5) The most preferred method was “listening to podcasts” (n = 116; 38%). This was followed by ‘reading on my own’ (n= 56; 18.3%), simulation labs (n = 55; 18%) and ‘small group discussions’ (n = 45; 14.8%). Overall, lectures, attending courses, and standardized patient encounters received significantly lower ratings.

**Table 5:** Preferred method of learning in residency

**Learning Method**	**N=305**	**Percentage**
Listening to Podcasts	116	38.0
Reading on my own	56	18.3
Simulation Labs	55	18.0
Small Group Discussions	45	14.8
Lectures	23	7.5
Attending Courses	8	2.6
Standardized Patient Encounters	2	0.6

## DISCUSSION

Our study results revealed that most respondents did not consider themselves completely comfortable in multiple communication scenarios, and that many residents felt that their training in patient-centered communication had been lacking. Given the feedback that we have anecdotally received during recent years, we also concluded that there is likely no standard way for educating EM residents in PCC and some residents appear to be receiving more training.

On average, dealing with difficult family members and/or friends had the lowest rated resident comfort level of the five scenarios. Residents rated themselves as second least comfortable in handling situations involving drug seekers. Our findings are consistent with previous studies that have demonstrated resident discomfort in managing chronic pain patients and prescribing pain medications.^9^ Although the Accreditation Council for Graduate Medical Education (ACGME) requires training in pain management, it continues to be an area in which many sample residents felt they had been inadequately trained. Interestingly, this was the second lowest rated area in regards to training. The scenarios regarding refusal of care by high-risk patients and giving bad news fell in the middle of the five scenarios in both comfort level and training differences.

As a whole, residents trended towards being very comfortable with exhibiting empathy, which was the highest rated of all five scenarios. Although not statistically significant, this was the only area (exhibiting empathy) in which junior residents were more comfortable than senior residents were. Anecdotal evidence in other settings has supported the belief that EM residents begin residency feeling more empathetic towards their patients than when they graduate.^10–12^ Ideally, most junior residents will develop these types of interpersonal skills during their progression through residency.

### Training Improvements

EM Residency program faculty may want to consider ways to provide their residents with more training in how to handle these types of challenging situations and improve their communication practices toward patients. These results indicated that some EM residents may use podcasts, independent reading, and simulation labs as their preferred learning methods in these areas.

### Study Limitations

Though content from the EBEM EM Resident Milestones were utilized in creating the survey questions in this study, the survey itself was not validated. In addition, the amount of training that respondents reported was subjective and it is difficult to know how many hours/what type of training the residents received in any of these areas of practice. It is certainly unclear whether respondents’ actual competence was related to their reported comfort levels and/or attainment of ACGME communication milestones. Finally, our results may have been skewed from this self-selected convenient sample.

## CONCLUSIONS

In summary, training in communication/difficult situations faced in the ED appears to be lacking in many EM residency programs, contributing to lower comfort levels for many EM residents. However, many of our sample respondents perceived themselves as being quite comfortable exhibiting empathy and giving bad news to patients. It is interesting that comfort in giving empathy in our sample was the only skill that appeared to diminish during the later years of residency. Given the importance of having strong communication skills as an EM physician, it should be made a priority by residency programs to establish curricula that help residents further develop these skills and achieve ACGME milestones prior to graduation.

### Conflict of Interest

The authors declare no conflict of interest.
